# Wound of the Heart

**Published:** 1835

**Authors:** R. P. Simmons

**Affiliations:** Cincinnati


ART. II.—WOUND OF THE HEART.

*Case of a Gun Shot Wound of the Heart.* Reported by R. P. SIMMONS, M. D. for the Western Medical Journal.

On the 21st December, about five o'clock, P. M. an affray took place, between Charles F. Gedney and Isaac Maguire, in which the latter was shot in the breast with a small pocket pistol, loaded with a bullet, of the size of seventy-four to the pound.

No evidence of a mortal wound was perceived for the moment, and Maguire, with strength and agility, pursued his antagonist, in his attempts to escape, for some rods, when he became faint and fell. At this stage of the catastrophe Dr. Whitman saw Maguire, dressed the wound, and ordered the necessary management for the time.

I was then requested to visit, and take charge of the case, which I promptly did, and saw the patient for the first time, about two hours after the occurrence.

On examining the wound, it was found that the ball had entered the body, between the sixth and seventh left ribs, at their junction with the sternum, and on making attempts to ascertain its direction, I was soon satisfied that it was beyond the reach of extraction.

The symptoms presented at this time, were referable, not only to some important organic lesion of the viscera of the chest, but also indicated a sympathetic or secondary affection of the brain. The temperature of the skin was very much below the natural degree, and it exhibited a livid appearance, particularly about the face and lips — no pulse was perceptible in any position of the arm, nor even in the carotids — there was excessive vomiting, accompanied with two or three free and copious evacuations from the bowels, the patient raising from his bed to the stool with surprising strength and activity. About this time a short but perceptible rigor came on, succeeded in a little while by pulsation in the arteries, and an elevation in the temperature of the body. The mind suffered from severe apprehension, but the patient was unable to look back upon the scene, or to have the most indistinct conception of what had transpired; he made frequent and importunate inquiries, to know how, where, by what hand, and for what cause, he was thus prostrated, and when as often informed, was still unable to associate the present with the past circumstances in this unfortunate occurrence.

The only *therapeutic* measure instituted at this time, was Tinct. Opii ʒss, with a little brandy. and water, to be given *pro re nata*, with warm applications to the extremities, and the injunction of a quiescent posture of the body.

22d instant, eight o'clock: — visited the patient this morning. Pulsation had fairly returned to the wrists, numbering only hundred in the minute, and affording some expectation that re-action would become established. The temperature of the body considerably elevated, but great paleness of the face, livid lips, white tongue, great thirst, some pain and *distress* in the chest, and was unable to lie in any other position than on his back. When no attempts were made to change his posture, his respiration was easy, and the pulse more regular and free in its beats. Mind still unsatisfied, and had no recollection of any thing that had happened.

Prescribed the strictest antiphlogistic regimen. At one o'clock, P. M., appearances about as they were at our former visit, except a little more acceleration in the circulation, the pulse, numbering one hundred and five.

Five o'clock, P. M., four hours after, saw the patient, and notwithstanding ℥xxx of blood had been drawn from the arm during the *interim*, by Dr. W., there was about the same force in the circulation as at our former visit. Pulse one hundred and five, regular, and quick in its beats. Temperature a little above that of health, thirst excessive — lividness of the face and lips the same — great pain and *distress* in the chest and epigastrium. Prescribed a gentle laxative, and acidulated drinks.

At nine o'clock, P. M., saw our patient, when we met Doctor Whitman for the first time, who had been requested to continue his attendance, as *consulting physician.*

No very perceptible change in the condition of the patient. Pulse one hundred and ten, regular, quick, and perhaps communicating to the fingers a little more wiry *jerk.* Thirst unquenched — same painful uneasiness in the chest and epigastrium.

From the regular and gradual elevation in the powers of life from the time that action was restored to the heart and arteries, till the present, we could arrive at no other conclusion, than that the case would terminate in inflammation; our object, therefore, was to obviate, if possible, suppuration or *gangrene.*

Took about ℥xvj of blood, and prescribed Calomel gr. xv., Pulv. Dov. gr. viij., Anti. Pulv. gr. iij., to be be taken at one dose.

23d inst. — Saw the patient this morning at half past eight o'clock. Rested pretty comfortably through the night; had two hours undisturbed sleep; pulse still quick in its beats, a little hard, but without much force, and one hundred and five in the minute. Thirst intense; countenance pale; lips livid; tongue and gums white; no mitigation of the pain and *distress* in the chest. Had no evacuations during the night. Mind still unsettled. Prescribed Sulph. Magnes. ℥j., Tart. Antim. gr. j., mixed and dissolved in one pint of water; one table spoonful to be taken every two hours.

Two o'clock, P. M. — Patient had taken twice of the saline mixture, was considerably nauseated, and vomited two or three times, when he discontinued the medicine. The symptoms of pain, restlessness, thirst, &c., not in the least degree mitigated. Pulse one hundred and ten, quick, and a little hard. Took about ℥xx of blood, and ordered the Sal. Mix. to be taken in smaller doses.

Seven o'clock in the evening, in consultation with Dr. W., found the patient about in the same state as when we left him five hours previously, except that the pulse had less force and frequency. No evacuations from the bowels during the day and night previous. Prescribed Ep. Sal. and Senna in small doses.

24th inst. — Nine o'clock, A. M., saw the patient agreeably to appointment. The medicine had operated three times during the night, unloading the bowels of much feculent and bilious matters, still there was no amelioration in his condition. The same painful *uneasiness* in the chest was complained of. Thirst unquenchable. Pulse one hundred and five. Ordered diluents and Emp. Canth. to be applied to the chest.

Three o'clock, P. M. Visited the patient. Pulse one hundred, and communicating to the fingers a very peculiar jerk in its beats. Respiration, for the first time we noticed it, a little uneasy. Antiphlogistic treatment continued.

At seven o'clock, the usual hour for our evening consultation, we again visited our patient, and to state the symptoms would only be to repeat what has been more than once repeated, during the course of this narration. Prescribed Cal. ℈j., Pul. Dov. gr. x., Antimo. Pulv. gr. iv., mixed and divided into three papers — the first to be taken at eight o'clock, the second and third as occasion might require.

At eleven o'clock was called on to visit the patient in great haste. Found him laboring under violent efforts to vomit, with increased pain in the chest and epigastrium. Prescribed Sulph. Morph. gr. ss., in six pills — one to be taken, and repeated if relief were not obtained.

25th inst. A. M. — Sickness alleviated in some degree. Pulse intermittent, and feeble; countenance distressed and cadaverous; thirst still the first and the greatest complaint; some pain, and vomited when more than one spoonful of drink was taken. Ordered wine whey, with sinapisms to the extremities.

Twelve, M.—Pulse scarcely perceptible, and continued to recede, with a corresponding dimunition in all the powers of life, till seven o'clock, P. M., when death in its *mildest form*, terminated the scene, being ninety-seven hours after the wound was inflicted.

*Post obit examination.*—Fifteen hours after death we proceeded to the examination of the body, by making an incision from the superior part of the sternum, and as far down on either side of the abdomen as the lumbar region. The abdominal muscles were then turned aside, and a general view taken of the abdominal viscera, and lower surface of the diaphragm, all of which presented a healthy aspect. The integuments, and muscular covering were now dissected from the ribs and sternum, and we found, as before stated, that the ball had entered the thorax between the sixth and seventh ribs, at their attachment to the sternum. There was considerable extravasation around the wound, and the tip of the cartilage of the sixth rib was detached from the sternum. The cartilages of the ribs, and sternum were then removed, bringing into view the pleura and anterior mediastinum, both structures exhibiting strong marks of inflammation. A little to the right, and exactly corresponding to the external wound, was traced the passage of the ball through the mediastinum, the whole anterior portion of which was carefully removed, with the adhering portion of the pericardium. The latter membrane was much injected, and its whole internal parieties completely covered with a continuous layer, resembling very much in appearance the deciduous membrane of the gravid uterus.

This adventitious membrane, lining the internal surface of the pericardium, and giving a complete envelope to the heart, was so firm and resisting in its texture, that it required considerable force to separate it.

The heart was next examined, which appeared in its natural position, and the pericardium and adventitious membrane being removed, there appeared on its anterior and right lateral surface, about two and a half inches from its apex, a small indentation, resembling very much the eschar of a recently healed wound. A ligature was now applied to the vena cava, and the heart removed from the body. At first sight it did not appear that there was any solution of continuity in this organ; but when a small blunt pointed probe was slightly pressed on the centre of the wound, it readily passed into the right ventricle. The ventricle was now laid open, and on examining its internal parieties, the wound exhibited a more open and lacerated aspect. Thus far in our examination the ball was clearly traced, and we were well satisfied that it had penetrated the right ventricle, but as it could not be found there, nor any marks of its forcible exit presenting, we were unable, for a time, to account for the phenomenon. The right auricle was next opened, and its internal structure examined, without affording any signs of a single fibre having been wounded, in the slightest degree.

We will here notice, that both the right auricle and right ventricle were nearly filled with coagulable lymph, which extended into the vena cava ascendens, some distance.

There was no blood found in the cavity of the thorax, nor within the pericardium.

The heart was macerated a short time, when T. P. Hotchkiss, one of my students, on examining, noticed a small wound in the centre of the tricuspid valves, which was confirmed by all present and afforded a satisfactory solution to the problem.

It was now so natural to conclude that the ball had descended into the vena cava ascendens, that it would have been denying the very first law of physics to have doubted. The vein was accordingly explored by Dr. W., as far down as its bifurcation when, the ball was detected, fairly within the right iliac branch. Thus, the ball entered the right ventricle of the heart, passed to the right auricle, through the tricusped valves, and then by its gravity descended into the vena cava ascendans and was found lodged in the right iliac portion, about half an inch from its bifurcation.

Although the annals of Medical Surgery, do not perhaps furnish a parallell to this truly novel case, I am nevertheless aware, that there have been many instances of more extensive gun shot, and other wounds of the heart, when death was not the immediate consequence. One of these and perhaps the most extraordinary case on the pages of medical record is to be found in Dorsey's elements of Surgery, as related by Dr. Babington, where a marine on board a ship of war received a bayonet wound of the heart and lived nine hours.

"Upon dissection it was found that the bayonet had penetrated through the integuments, the abdominal muscles, and peritoneum, had pierced the colon, the stomach, the left lobe of the liver, the diaphragm, and indeed the thorax at its centre. Immediately within the breast, the pericardium had presented and yielded the instrument a ready passage to the heart. The right ventricle then received it. The point was thrust in at the lower part of the ventricle and had forced its way out near the valve. From the heart again, passing the pericardium, it pierced through both the upper and middle lobes of the lungs, but even these were insufficient to detain it. It forced a passage on the right side, near the sternum between the cartilages of the second and third ribs, and had sheathed its point beneath the pectoral muscle. That muscle was slightly wounded, but the integuments above it were unhurt. A little bloody serum was found in the cavity of the belly, but scarcely any pure blood, the pericardium contained a little blood. The right side of the heart contained above two quarts of blood, partly in a fluid, and partly in a congealed state."

*Cincinnati, January 1st,* 1836.

